# Late kidney transplant dysfunction related to proximal aorto-iliac occlusive disease

**DOI:** 10.3389/ti.2026.15946

**Published:** 2026-08-20

**Authors:** Eva De Vis, Maarten Naesens, Ina Jochmans, Kathleen Claes, Annouschka Laenen, Diethard Monbaliu, Mauricio Sainz Barriga, Annelies Laerte, Dirk Kuypers, Jacques Pirenne, Geert Maleux

**Affiliations:** 1 Department of Radiology and Department of Imaging and Pathology, University Hospitals KU Leuven, Leuven, Belgium; 2 Department of Nephrology, University Hospitals KU Leuven, Leuven, Belgium; 3 Department of Abdominal Transplant Surgery, University Hospitals KU Leuven, Leuven, Belgium; 4 Department of Biostatistics and Statistical Bioinformatics, University Hospitals KU Leuven, Leuven, Belgium

**Keywords:** angioplasty, dysfunction, outcome results, proximal aortoiliac occlusive disease, stent

## Abstract

This study is a retrospective analysis of a cohort of patients presenting with transplant renal dysfunction related to proximal aorto-iliac occlusive disease and treated with angioplasty and stenting. Primary outcomes included changes in intrarenal resistive index (RI) and laboratory and clinical data including serum creatinine level, systolic and diastolic blood pressure, and number of anti-hypertensive drugs before and after endovascular intervention. Secondary outcomes included overall patient and transplant kidney survival. Endovascular intervention was successful in all 26 included patients and associated with a significant increase in intrarenal RI in the upper pole (0.65 before vs. 0.74 after stent; P = 0.0009), in the interpolar region (0.64 before vs. 0.74 after stent; P = 0.0028), and in the lower pole (0.63 before vs. 0.76 after stent; P = 0.0002). In addition, there was a significant improvement in serum creatinine (2.29 mg/dL before vs. 1.49 mg/dL 6 weeks after stent; P < 0.0001) and systolic blood pressure (149.6 mmHg before vs. 139.8 mmHg 1 week after stent; P = 0.0233). Overall estimated transplant kidney survival was 95% at 5 and 10 years of follow-up. Endovascular intervention is a very effective and durable treatment option for the management of transplant renal dysfunction related to proximal aorto-iliac occlusive disease.

## Graphical Abstract

**Figure:**
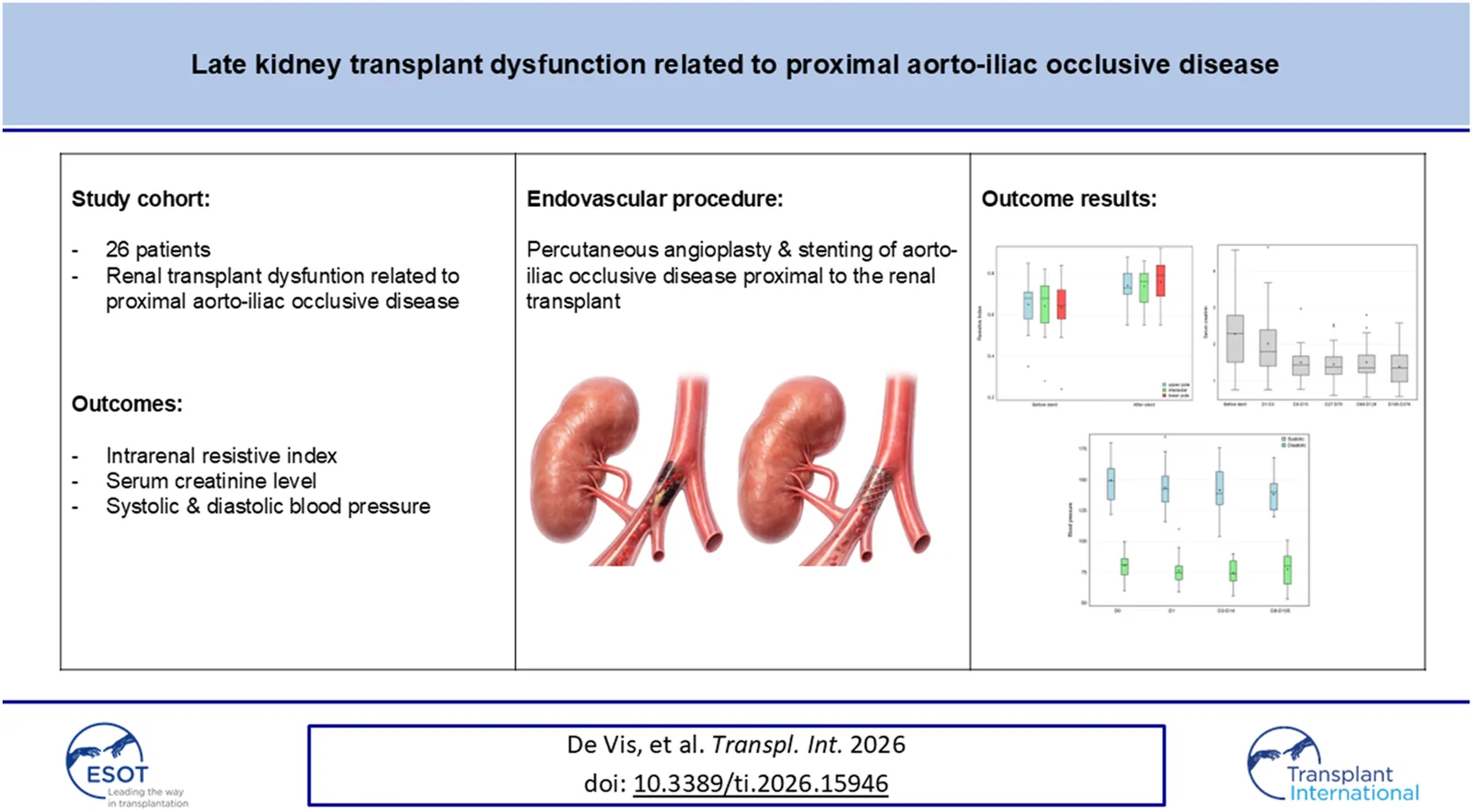

## Introduction

Renal transplantation is a cornerstone treatment for patients with end-stage renal disease, significantly enhancing their longevity and quality of life [1]. Nevertheless, there is a low but notable risk of early and late post-kidney transplant vascular complications, with an incidence of 3%–15% [2, 3]. Vascular arterial complications include the formation of intrarenal pseudoaneurysms or arteriovenous fistulas, often related to posttransplant renal biopsy and transplant renal artery stenosis (TRAS) or aorto-iliac stenosis proximal to the kidney transplant artery (pTRAS) [2, 3]. Potential consequences of (p)TRAS include a decline in kidney transplant function, renovascular hypertension, and ultimately graft loss. Open surgical repair to treat (p)TRAS has been associated with high morbidity and risk of graft loss [4]. Endovascular interventions are minimally invasive and well-tolerated by renal transplantation patients and are becoming increasingly used.

Several studies investigated the clinical outcome of patients with a TRAS treated with endovascular intervention, demonstrating the safety of this endovascular procedure and the associated clinical benefit in the majority of patients [2, 5].

In contrast, data on the outcome of patients with symptomatic, late aorto-iliac stenosis proximal to the transplant renal artery and treated with endovascular intervention are scarce and mostly limited to case reports [6–9] and small-size observational studies [10, 11] with a limited number of patients, observed over a limited follow-up period. Therefore, we conducted a retrospective cohort study including patients with transplant kidney dysfunction caused by aorto-iliac occlusive disease proximal to the transplant renal artery who underwent endovascular intervention.

## Materials and methods

### Study design

This is a retrospective, observational study, including consecutive patients collected from the institutional interventional radiology database who underwent endovascular aorto-iliac intervention for the management of late transplant kidney dysfunction in the authors’ institution from January 2005 till August 2022. Patients’ demographic data and pre-and post-interventional laboratory and clinical data were collected from the patients’ electronic medical records. Patients’ cardiovascular risk factors were summarized based on Rutherford’s standards for lower extremity ischemia [12].

The institutional Ethics Committee approved this retrospective study (MP 022096).

### Baseline clinical and laboratory evaluation

An upstream aorto-iliac stenosis was considered as symptomatic if the patient presented with a concomitant serum creatinine decrease of >30% over a time period of less than 6 months and/or if the patient presented with a progressive and resistant increase in arterial blood pressure up to 150/90 mmHg or more over a time period of less than 6 months, despite administration of additional antihypertensive drugs if supported by the patient.

### Duplex ultrasound with resistive index (RI) measurement

The arterial RI was measured in the upper pole, mid-portion, and lower pole of the transplant kidney; RI calculation was performed according to Naesens et al., as [1-(V_min_ ÷ V_max_) ] with V_min_ representing the end-diastolic velocity and V_max_ the peak systolic velocity [13]. An RI in renal transplants is within normal limits between 0.6 and 0.8; <0.6 and >0.8 were defined as significantly low and significantly high RIs, respectively [11, 13]. Further, the intrarenal Doppler waveform was also analyzed: a ‘tardus (slow) et parvus’ (little) waveform reveals a dampened flow and can be used as an indirect sign of a proximal vascular stenosis.

### Computed tomography angiography (CTA)

CTA was performed in patients with an acceptable, residual renal function (serum creatinine <1.2 mg/dL). The arterial phase was performed after intravenous injection of 80 mL of iohexol iodized contrast medium (Omnipaque 350, GE Healthcare, Oslo, Norway) at a flow rate of 4 mL/s and when the automated triggering in the proximal abdominal aorta reached 100 Hounsfield units.

### Magnetic resonance angiography (MRA)

Contrast-enhanced MRA was performed after intravenous injection of gadobutrol 0.1 mL/kg (Gadovist, Bayer AG, Berlin, Germany) at a dose of 0.5 mmol/mL and a volume based on the formula patient weight (kg) x 0.2. A care bolus injection was also performed to determine the optimal arterial phases.

### Technique of the interventional procedure

After obtaining the patient’s informed consent, the contralateral common femoral artery was punctured under local anesthesia and a 55 or 65 cm long, 6 or 7 French (F) sheath (Flexor, Cook Medical, Bloomington, IN, USA or Arrow sheath, Teleflex, Morrisville, NC, USA) was placed. Angiographic mapping of the aorto-iliac vessels was performed with iohexol iodized contrast medium (Omnipaque 270, GE Healthcare, Oslo, Norway) and/or carbon dioxide (CO2) (Angiodroid, CO2-injector, San Lazzaro di Savena, Italy or CO2-Angioset, Optimed, Ettlingen, Germany). The iliac stenosis was defined as occlusion, high-grade stenosis (>90% stenosis), intermediate grade stenosis (70%–90% stenosis), or low-grade stenosis (<70% stenosis). After an intra-arterial administration of 5000 international units of heparin, the stenosis was retrogradely cannulated and predilated with a 6 mm standard angioplasty balloon (Admiral Xtreme, Medtronic, Minneapolis, MN, USA and Mustang, Boston Scientific Inc, Cork, Ireland); in case of vessel wall recoil, a balloon-expandable stent was placed in the common iliac artery. Depending on the time period, various types of stents were used including Cordis Palmaz-Genesis (Cardinal Healthcare, Miami Lakes, FL, USA), Dynamic, [Biotronik, Berlin, Germany), Express Vascular (Boston Scientific Inc, Natick, MA, USA), Scuba Invatec (Medtronic, Minneapolis, MN, USA); in case of an aortic stenosis, a covered balloon-expandable stent (CP-stent, Numed, Hopkinton, NY, USA) was inserted and, in case of a stenosis at the distal end of the common iliac artery or proximal external iliac artery, a self-expanding nitinol stent (Zilver, Cook Medical, Bloomington, IN, USA) was inserted. Finally, if an additional stent in the transplant renal artery was needed, a balloon-expandable renal stent (Tsunami, Terumo Europe, Leuven, Belgium) was used. After completion of the angiography, the puncture site was closed with a closure device (Angioseal, Terumo Europe, Leuven, Belgium) and the patient was prescribed 80 mg daily aspirin lifelong.

### Follow-up and study endpoints

Patients were followed up by the attending nephrologist; the study follow-up period ended in January 2024. Primary outcome data included changes in RI, in serum creatinine levels, in systolic and diastolic blood pressure, in number of administered antihypertensive drugs, and in daily dose intensity (DDI) for antihypertensive drugs, measured according to Min et al. [14]. Secondary endpoints included transplant kidney and overall patient survival.

Duplex-ultrasound with measurements of the RI were performed 1 week, 1 month, 6 months, and yearly after the index endovascular procedure. Laboratory analysis and clinical evaluation were performed at each follow-up visit; for comparative analysis, serum creatinine levels were collected before and 1 and 3 days after intervention as well as 1 week and 2, 3, and 9 months after the index endovascular procedure. Blood pressure measurement and number of prescribed antihypertensive drugs were collected at 1 week, 1 and 6 months, and 1 year after the index endovascular procedure.

### Statistical data analysis

Descriptive statistics were used to characterize the demographic data of the study population.

Linear mixed models for longitudinal measurements were used to analyze the effect of endovascular intervention on blood pressure, serum creatinine level, the number of used antihypertensive drugs, and the intrarenal RI of the transplant kidney. A random intercept was modelled to account for data clustering. Results are reported as mean with 95% confidence intervals and mean differences to compare follow-up measurements (after endovascular intervention) with measurements before the endovascular intervention.

Kaplan Meier estimates were used to estimate the kidney failure rate over time, accounting for death as a competing event. Analyses have been performed using SAS software (version 9.4 of the SAS System for Windows, Cary, NY, USA).

## Results

### Patient characteristics

In this study, 26 consecutive patients (14 male and 12 female patients) with a mean age of 53.4 ± 12.7 years were included. One patient treated 3 months after kidney transplantation and presenting with two focal external iliac artery stenoses, proximal and distal to the origin of the transplant renal artery and related to a vascular clamp injury during transplantation, was excluded for further analysis. In the same study period, no institutional patient with identical radiological and clinical characteristics and symptoms was managed with conservative or open surgical techniques; in addition, an extensive search of transplant patients’ medical records, followed up in the authors’ institution before 2005, could be identified, making a comparative outcome analysis impossible. Fourteen out of the 26 included patients (54%) were transplanted between January 2000 and September 2022; in the same time period, 2286 kidney transplantations were performed in the authors’ institution. Patients’ baseline demographics and clinical data are summarized in Table 1.

**TABLE 1:** Patients’ cardiovascular risk factors and kidney transplant data.

Cardiovascular risk factor	N (%)
Diabetes Adult onset, controlled by diet or oral agents Adult onset, insulin-controlled Juvenile onset	7/26 (27%) 5/26 (19%) 2/27 (8%)
Tobacco use Not current but smoked in last 10 years Current smoker	6/26 (23%) 4/26 (15%)
Hypertension Requires more than two drugs or is uncontrolled	2/26 (85%)
Hyperlipidemia Mild elevation, readily controllable by diet Requiring dietary and drug control	5/26 (19%) 14/26 (54%)
Cardiac status Myocardial infarction by history (>6 months) Stable angina or no angina but significant reversible perfusion defect	11/26 (22%) 8/26 (31%)
Carotid disease Transient or temporary stroke Complete stroke with permanent neurologic deficit	1/26 (4%) 1/26 (4%)
Renal status Kidney transplant Previous transplants Maintenance immunosuppression	26/26 (100%) 0/26 (0%) 26/26 (100%)
Pulmonary status Vital capacity less than 1.85 L	1/26 (4%)
Kidney transplant data mean median Std range Age (years) of donor at transplantation 53 56 13 (22; 70) Warm ischemia time (minutes) 31 30 9 (20; 44) Cold ischemia time (minutes) 680 751 319 (297; 1165)	
Total number of renal arteries 1 2 3	15/20 (75%) 4/20 (20%) 1/20 (5%)
Type of arterial anastomosis Arterial patch – EIA Arterial patch – CIA Donor renal artery – EIA Donor renal artery – CIA	19/22 (86%) 1/22 (5%) 2/22 (9%) 1/22 (5%)

EIA, external iliac artery; CIA, common iliac artery.

Maintenance immunosuppression: Mycophenolate mofetil (CellCept, Roche) 1000 mg/day + methylprednisolone (Medrol, Pfizer) 4 mg/day + tacrolimus (Prograft, Astelas Pharma) 6–8 mg/day.

The kidney transplantation of the included study patients was performed between June 1982 and April 2021. The mean and median time interval between kidney transplantation and endovascular aorto-iliac intervention was 109 ± 89 and 103 months, respectively (range 3–301 months). Details on the initial kidney transplantation and on patients’ clinical and radiological work-up are summarized in Tables 1, 2 respectively.

**TABLE 2:** Patients’ symptoms and pre-interventional imaging.

Patients’ symptoms	N (%)
Impaired renal function Impaired renal function and arterial hypertension Impaired renal function, arterial hypertension, & claudication Arterial hypertension Arterial hypertension and claudication Incidental duplex sonographic findings during protocol biopsy	5/26 (19%) 14/26 (54%) 3/26 (11%) 2/26 (8%) 1/26 (4%) 1/26 (4%)
Pre-interventional imaging Duplex ultrasound Duplex ultrasound & MR angiography Duplex ultrasound & CT angiography Duplex ultrasound, CT, & MR angiography CT angiography MR angiography X-ray angiography	7/26 (7%) 14/26 (54%) 1/26 (4%) 1/26 (4%) 1/26 (4%) 1/26 (4%) 1/26 (4%)

### Procedural data

**FIGURE 1:**
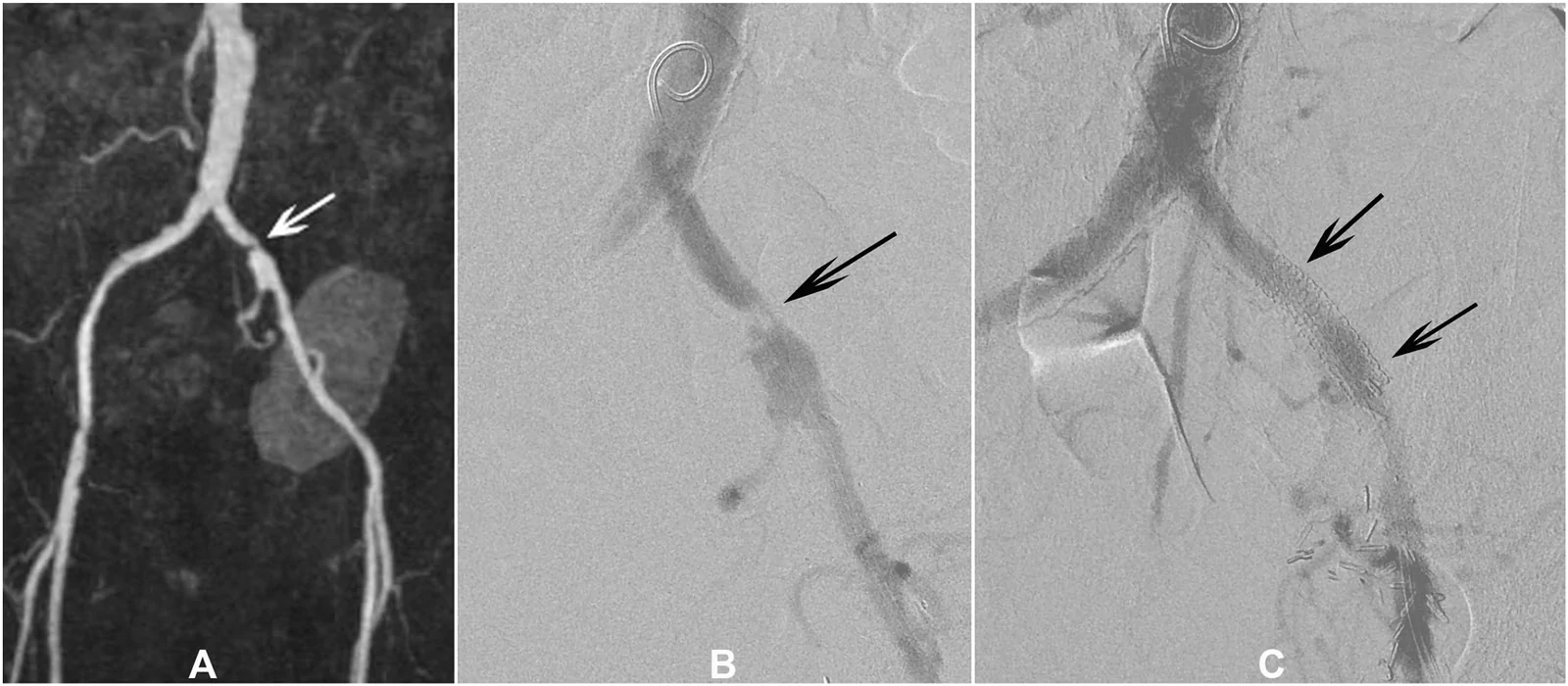
Hypertensive patient with serum creatinine decline (3.15 mg/dL) and focal, common iliac artery stenosis, proximal to the transplant kidney. **(A)** Magnetic resonance (MR) aortoiliac angiography shows a focal stenosis (arrow) of the left common iliac artery proximal to the transplant kidney. **(B)** Aorto-iliac angiography with use of carbon dioxide (CO2) reveals a focal stenosis of the left, distal common iliac artery (arrow), proximal to the transplant kidney. **(C)** Aorto-iliac angiography after stent insertion (arrows) shows restored patency of the left iliac axis.

**FIGURE 2:**
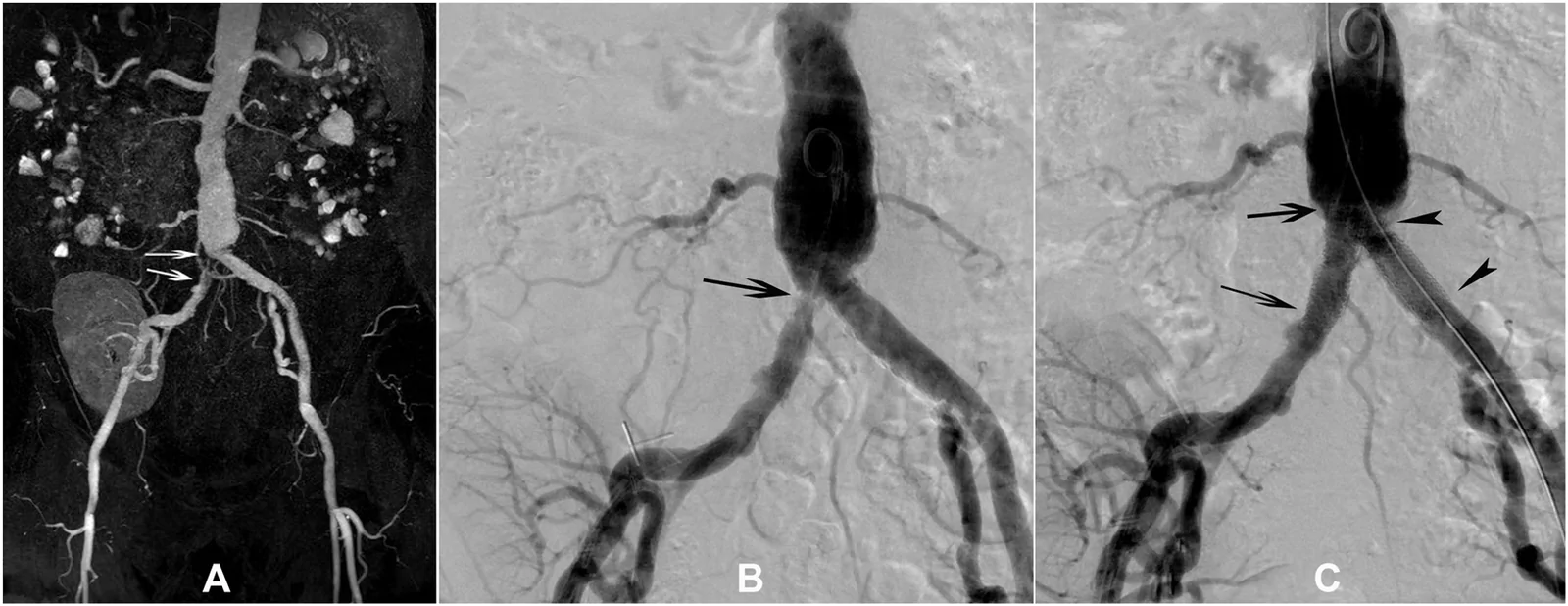
Hypertensive patient with an ostial common iliac artery stenosis proximal to the transplant kidney. **(A)** Magnetic resonance (MR) aortoiliac angiography shows a critical, ostial stenosis of the right common iliac artery (arrows) protruding into the aortic bifurcation. **(B)** Contrast aorto-iliac angiography shows the focal, ostial stenosis (arrow) protruding into the aortic bifurcation. **(C)** Post-stenting contrast-aortoiliac angiography shows fully patent kissing stents in the right common iliac (arrows) and left common iliac (arrowheads) arteries.

**FIGURE 3:**
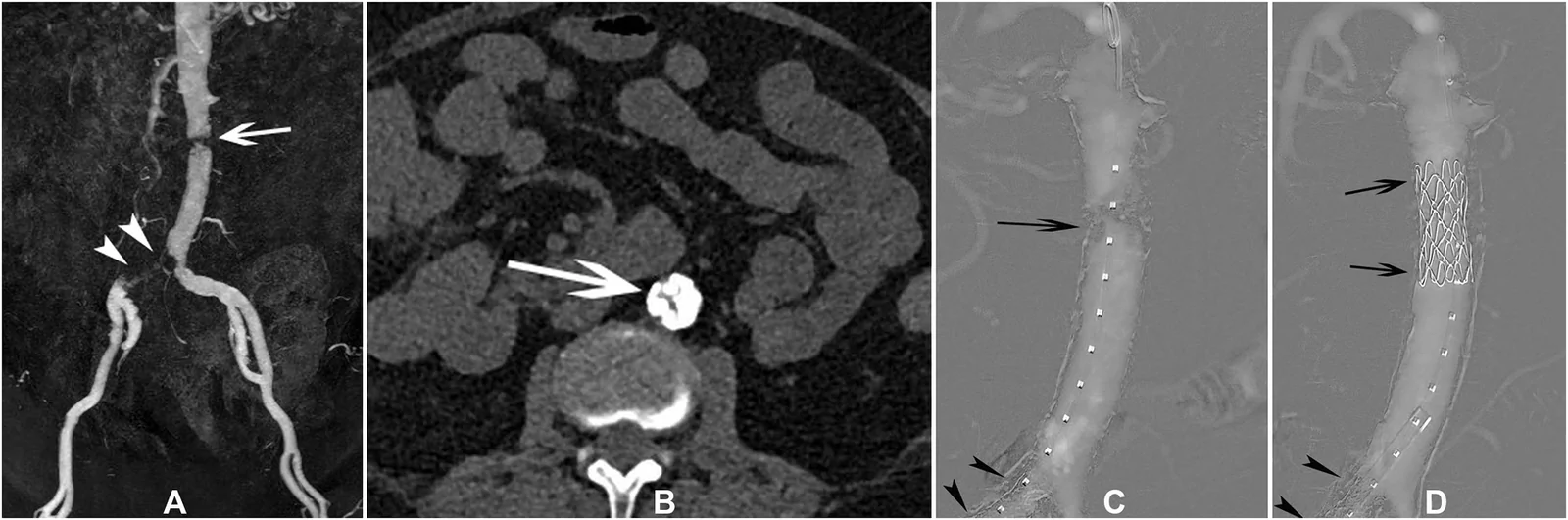
Hypertensive patient with serum creatinine decline (2.95 mg/dL) and focal stenosis in the distal aorta. **(A)** Magnetic resonance (MR) angiography demonstrates a focal, severe stenosis (arrow) in the infrarenal aorta; loss of signal in the right common iliac artery (arrowheads) due to stent artefact. **(B)** Unenhanced computed tomography confirms a circumferential and heavily calcified aortic stenosis (arrow). **(C)** Carbon dioxide (CO2) aortography reveals a focal, infrarenal aortic stenosis (arrow); note also the stent (arrowheads) in the common right iliac artery. **(D)** Carbon dioxide (CO2) aortography after endovascular intervention shows a fully expanded aortic stent (arrows); note also a stent in the proximal right common iliac artery (arrowheads).

Angiographic findings are summarized in Table 3 (Figures 1–3). All aorto-iliac stenoses were of atherosclerotic origin.

**TABLE 3:** Procedural data.

Characteristics of the aorto-iliac stenosis	N (%)
Severity of the stenosis Occlusion High-grade stenosis Intermediate-grade stenosis Low-grade stenosis	1/33 (3%) 30/33 (91%) 1/33 (3%) 1/33 (3%)
Length of the stenosis Mean Median Range	mm (millimeter) 12 11 (4; 20)
Location of the stenosis Infrarenal abdominal aorta Right CIA Right iliac bifurcation Right EIA Left CIA Left EIA Transplant renal artery	1/33 (3%) 12/33 (36%) 1/33 (3%) 2/33 (6%) 10/33 (30%) 4/33 (12%) 2/33 (6%)

Overall, technical success of the endovascular procedure was 100% and no procedure-related adverse events were reported.

### Ultrasonographic, laboratory, and clinical follow-up data

**FIGURE 4:**
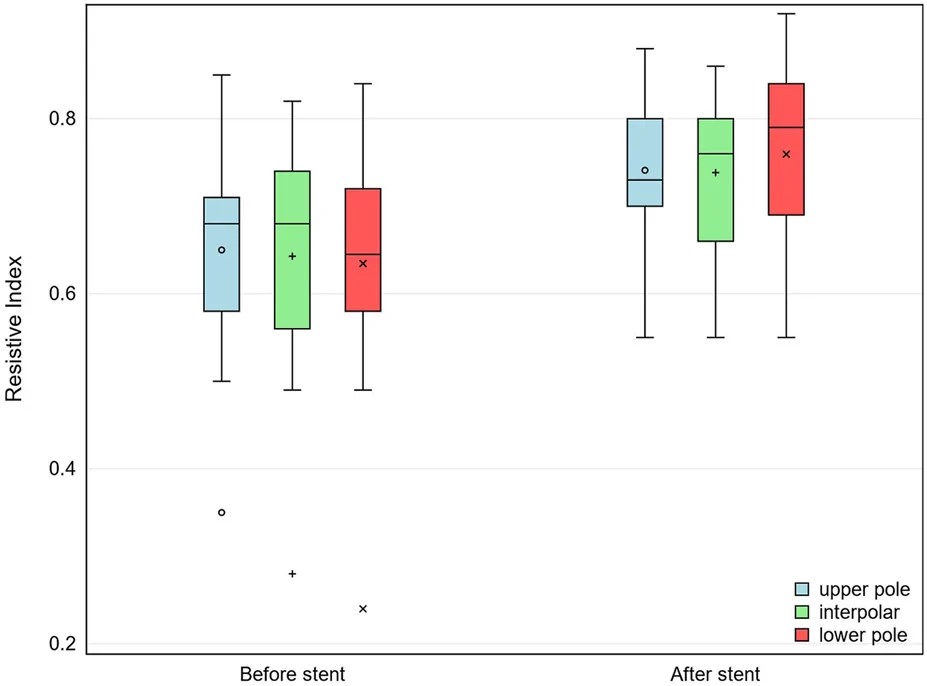
Box plot analysis of the difference in resistive index (RI) before and 1 week after aorto-iliac stent insertion measured in the upper pole, interpolar region, and lower pole of the transplant kidney.
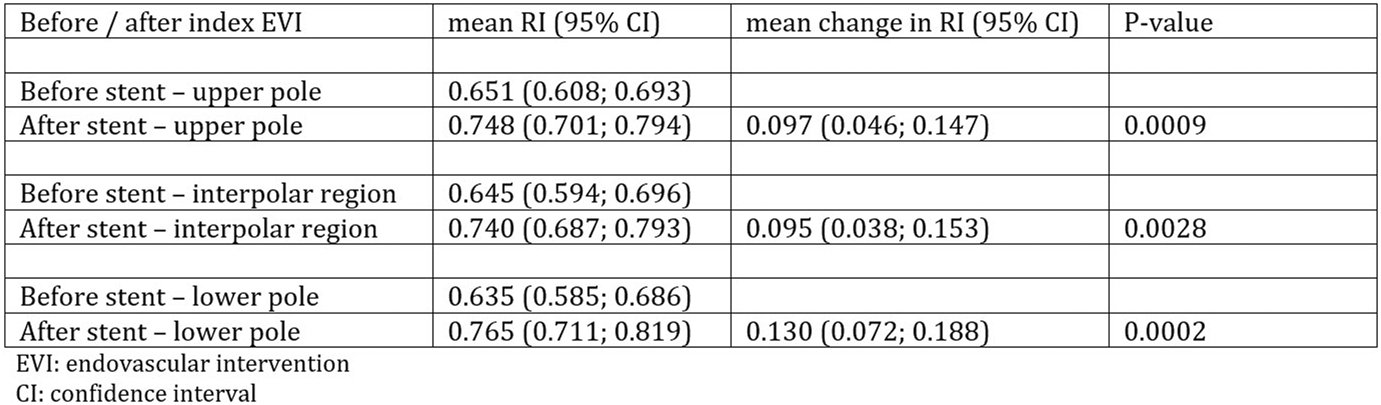

In the transplant renal upper pole, interpolar region, and lower pole, a significant rise in intrarenal RI of 0.097, 0.095, and 0.130 was found, as summarized in Table 4 and Figure 4.

**TABLE 4:** Intrarenal resistive index before versus after the endovascular procedure.

Region of interest	Before stent	After stent	P-value
Upper pole Mean Median Std Range	0.65 0.68 0.112 (0.35; 0.85)	0.74 0.73 0.079 (0.55; 0.88)	0.0009
Interpolar region Mean Median Std Range	0.64 0.68 0.131 (0.28; 0.82)	0.74 0.76 0.093 (0.55; 0.86)	0.0028
Lower pole Mean Median Std Range	0.63 0.65 0.131 (0.24; 0.84)	0.76 0.79 0.097 (0.55; 0.92)	0.0002

**FIGURE 5:**
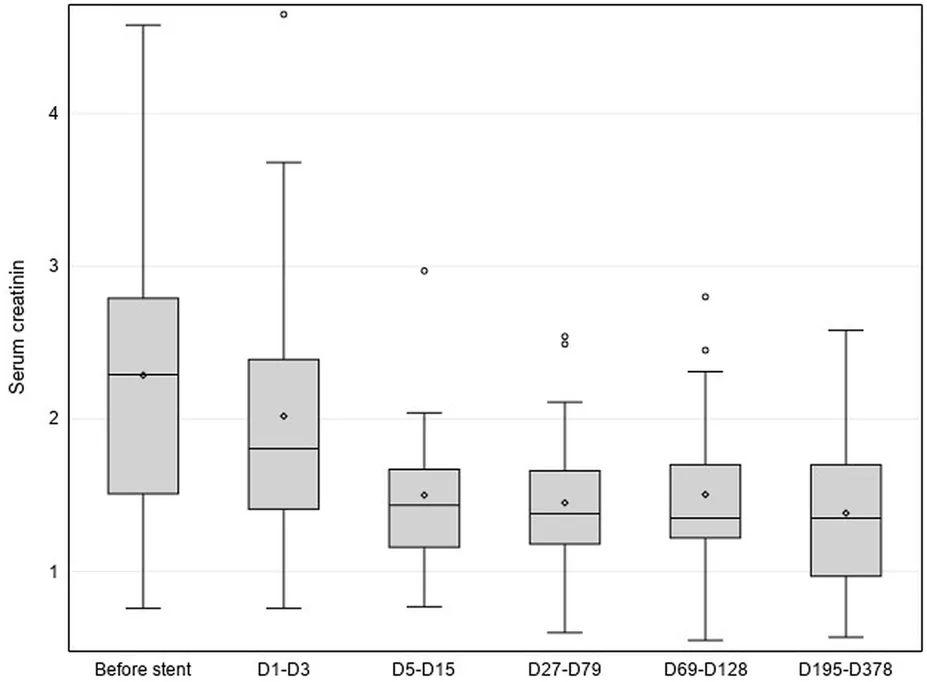
Box plot representation of the serum creatinine level (mg/dL), measured at different time points before and after endovascular intervention.
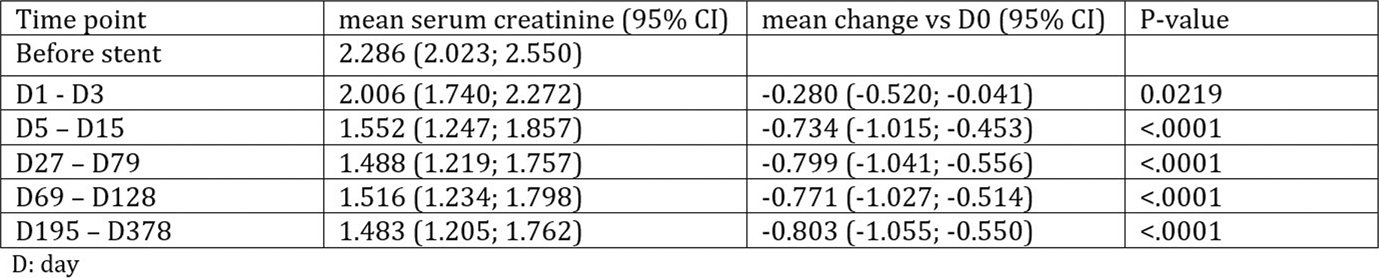

Serum creatinine levels before versus up to 1 year after the endovascular procedure are summarized in Figure 5, showing a significant and sustained decrease in values over time.

**FIGURE 6:**
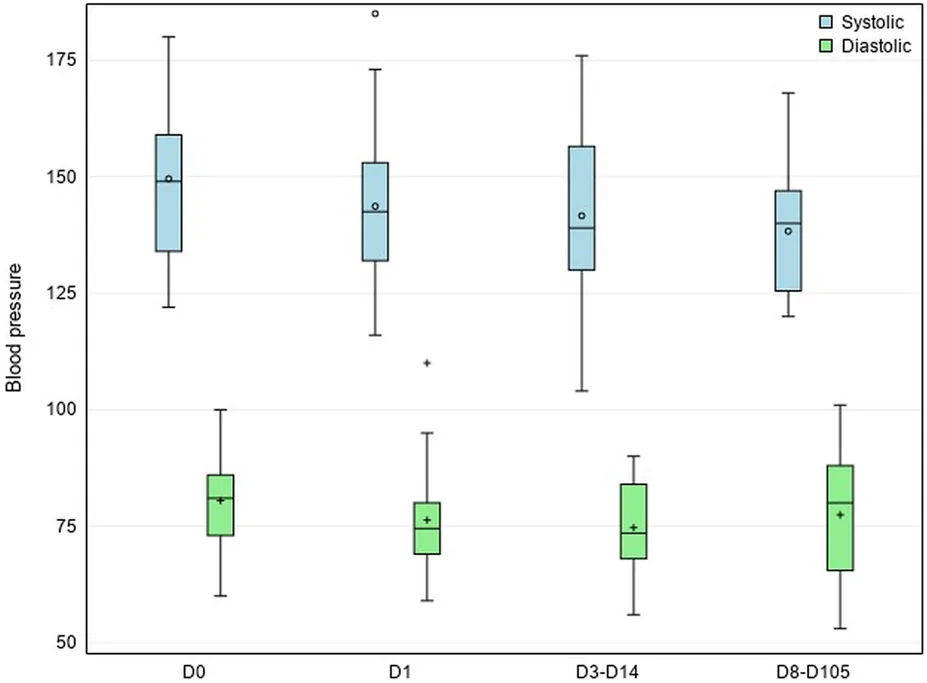
Box plot representation of the systolic and diastolic blood pressure (in mmHg) at different time points before and after endovascular intervention.
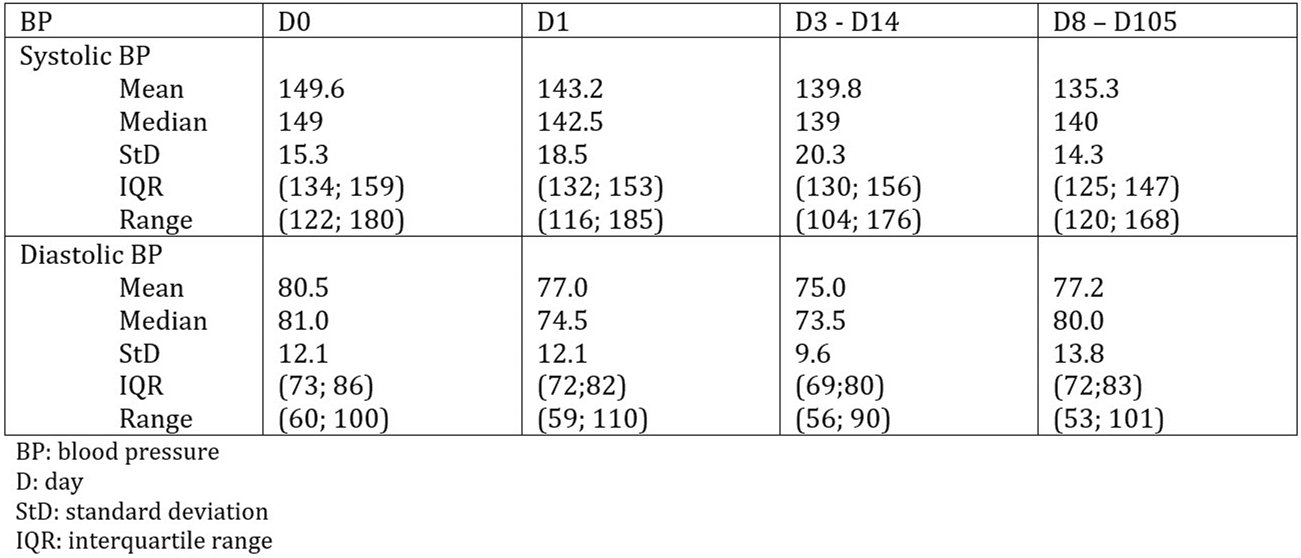

Analysis of the blood pressure before versus after the procedure revealed a significant drop in systolic pressure 1 week and 3 months after versus before the procedure; no significant drop in diastolic blood pressure over time was found, as demonstrated in Figure 6.

**FIGURE 7:**
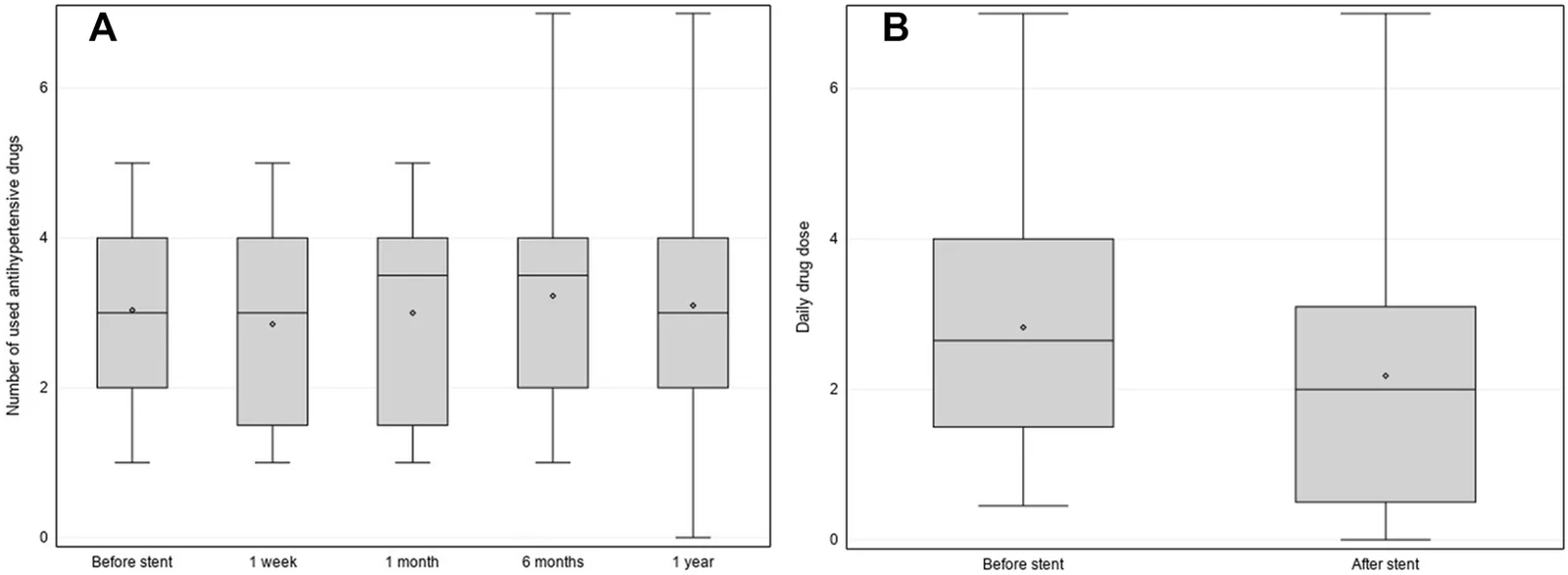
**(A)** Box plot representation of the number of used antihypertensive drugs at different time points before and after endovascular intervention. **(B)** Box plot representation of the daily drug dose (DDD) before the stent placement: 2.82 (2.13; 3.52, 95% confidence interval) versus: 2.18 (1.49; 2.87, 95% confidence interval) 6 months after stent placement, resulting in a mean change in DDD of −0.64 (−1.05; −0.24, 95% confidence interval) (P = 0.0030).
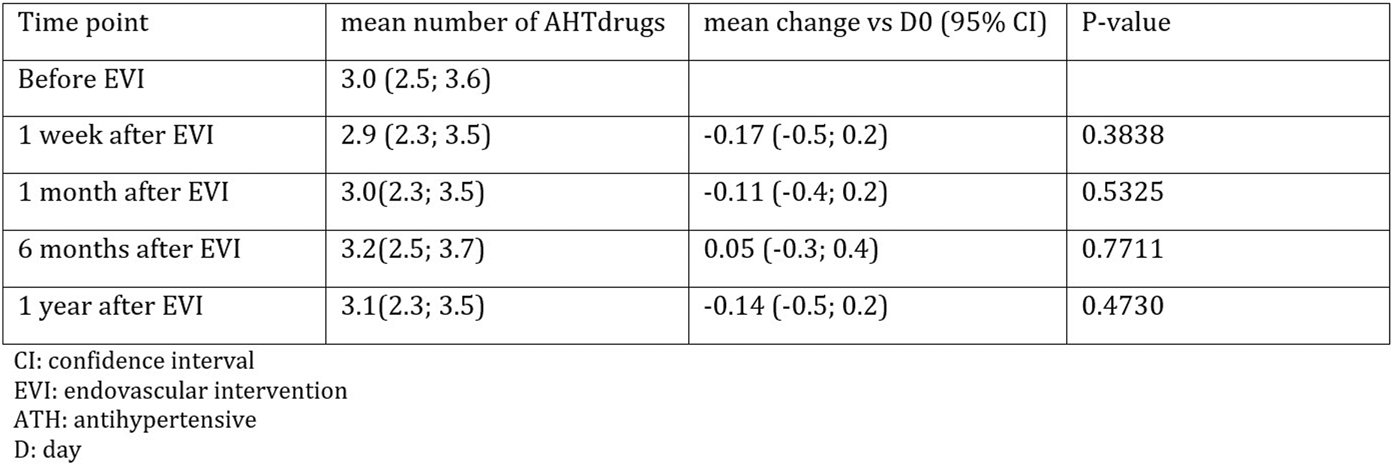

**FIGURE 8:**
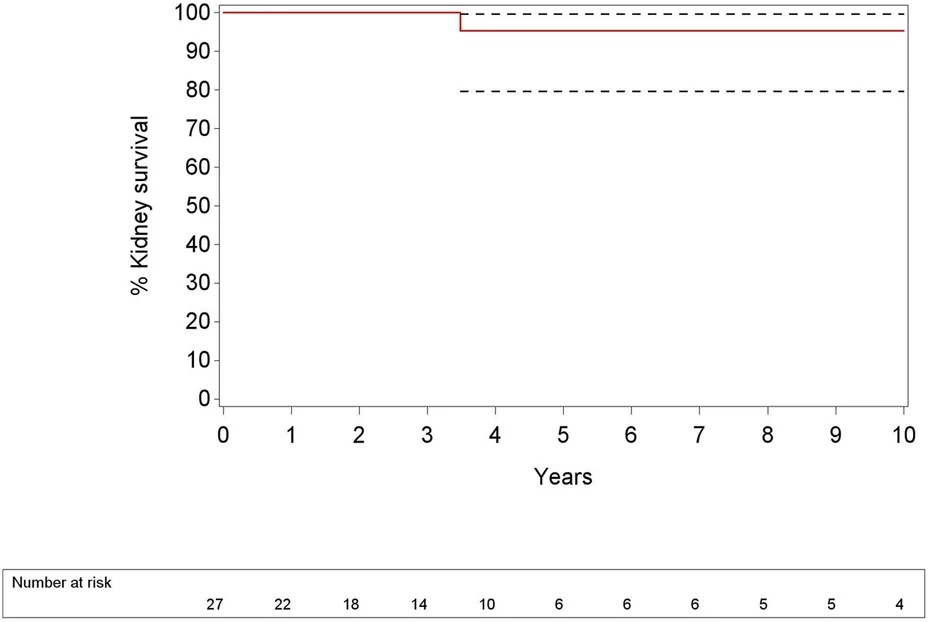
Kaplan-Meier analysis for transplant kidney survival. Kidney survival rates (95% confidence interval) show a 95.27% transplant kidney survival at 5 and 10 years of follow-up after aorto-iliac intervention respectively.

**FIGURE 9:**
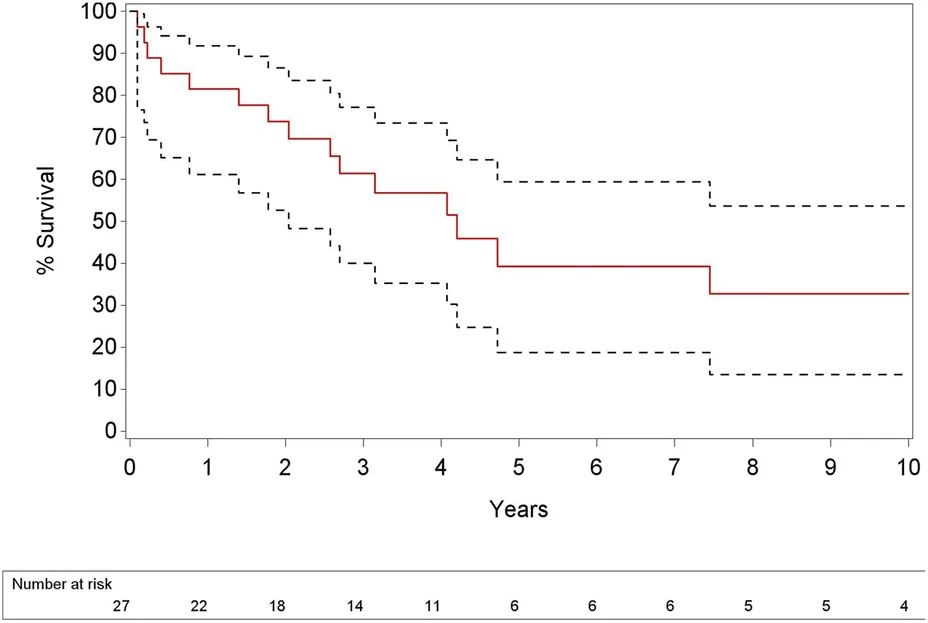
Kaplan-Meier analysis for overall survival after aortoiliac intervention proximal to the dysfunctional transplant kidney. Overall survival rates (95% confidence interval) show a median survival of 4.1 (2.0; 10.7) years after index aorto-iliac intervention. The overall survival estimates are 36 (15.6; 57) and 28.8 (10.3; 50.7) years at 5 and 10 years of follow-up after index aorto-iliac intervention, respectively.

Last, no difference in number of prescribed antihypertensive drugs before versus after the procedure was demonstrated, as summarized in Figure 7A; adversely, the mean antihypertensive daily drug dose before the stent procedure was 2.82 (2.13; 3.52, 95% confidence interval) versus 2.18 (1.49; 2.87, 95% confidence interval) 6 months after the stent procedure, resulting in a mean change of daily drug dose of −0.64 (−1.05; −0.24, 95% confidence interval; P = 0.0030) (Figure 7B). In the follow-up period after the endovascular procedure, one patient presented with a progressive transplant kidney failure and dialysis was started up 3 years after a successful CIA stenting, resulting in a transplant kidney survival of 95.0% (78.6; 99.7) and 95.0% (78.6; 99.7) at 5 and 10 years of follow-up respectively (Figure 8). In addition, 17 patients (65%) died during follow-up, resulting in a mean survival of 4.1 (2.0; 10.7) years with an estimated overall survival of 36.0% (15.6; 57.0) and 28.8% (10.3; 50.7) at 5 and 10 years of follow-up respectively (Figure 9).

## Discussion

This study reveals a very low incidence of 0.6% of pTRAS compared to reported early data ranging between 1.5% and 12.5% [2–4], most probably related to better screening and management of patients’ vascular status before and after transplantation. Potentially, the number of patients with pTRAS and associated transplant renal dysfunction might be underdiagnosed related to a more diffuse atherosclerotic involvement of one of both iliac arteries; in these patients, a chronic decline in renal function rather than a sudden rise in serum creatinine might be observed. Last, some patients might also be treated in other institutions despite index kidney transplantation and clinical follow-up in the authors’ institution.

From a clinical perspective, the majority of patients in this study were diabetic and hypertensive transplant patients with an onset of impaired kidney function after transplantation, which is in line with earlier data from Voiculescu et al., reporting a significant deterioration of hypertension (144 ± 15 mmHg systolic and 84 ± 9 mmHg diastolic at baseline versus 157 ± 22 mmHg systolic and 90 ± 10 mmHg diastolic immediately before intervention; P < 0.001) and increase of serum creatinine (1.7 ± 0.9 mg/dL at baseline and 2.5 ± 1.3 mg/dL) immediately before intervention [4, 11].

Duplex-ultrasound with RI measurements was diagnostic in the majority of cases, however, in 13% of patients, the proximal aorto-iliac stenosis was demonstrated by CTA/MRA only. Adversely, a tardus-parvus waveform was found in less than half of the patients.

Duplex-ultrasound with RI measurements seems to be a valuable tool for pTRAS screening: in 87% of patients, a low RI was found. Additional duplex-ultrasound evaluation of the common iliac artery, demonstrating a monophasic flow pattern, might be another sonographic, indirect sign for pTRAS [15].

Catheter-directed angioplasty and stenting of aorto-iliac occlusive disease has become the treatment of choice given the minimally invasive nature of the treatment, avoiding procedure-related morbidity and mortality and the current, high, and durable primary and primary-assisted patency rates [16], which is in contraindication to early reports demonstrating >50% restenosis after standard balloon angioplasty for pTRAS [11].

The clinical outcome of aorto-iliac stenting for pTRAS is favorable, with clear beneficial impact on renal function, which is in line with other reports on pTRAS [11] and TRAS [5]. In our study, a significant decrease in systolic pressure but not in diastolic pressure or in number of prescribed antihypertensive drugs was found, which contrasts with the findings of Voiculescu et al., who reported a drop in systolic and diastolic pressure and in antihypertensive medication [4].

Finally, this study has limitations. First, the retrospective study design might be associated with biased study results; second, the long interval of patient inclusion might influence clinical outcome results, in particular overall survival of patients treated early in the study might be different to patients treated more recently. In addition, the type of different stents used during the study period also changed; however, the technique of angioplasty and stenting did not change over time. Third, associated medical treatment, especially use and type of antihypertensive drugs, might be different throughout the study patients and potentially could have an effect on clinical outcome and patients’ overall survival.

In conclusion, endovascular management of pTRAS is safe, effective, and associated with a significant and durable increase in renal function and systolic pressure.

## Data Availability

The raw data supporting the conclusions of this article will be made available by the authors, without undue reservation.
